# Supplementary material for: A somato-cognitive action network alternates with effector regions in motor cortex
Source: Nature. 2023 Apr 19;617(7960):351–9. doi: 10.1038/s41586-023-05964-2 (PMC10172144; doi:10.1038/s41586-023-05964-2)
Supplement: Supplementary file 1 — This file contains Supplementary Tables 1 and 2 and Supplementary Figs. 1–8. [file 41586_2023_5964_MOESM1_ESM.pdf]

nature portfolio

https://doi.org/10.1038/s41586-023-05964-2

Supplementary information

A somato-cognitive action network alternates with effector regions in motor cortex

In the format provided by the authors and unedited

SUPPLEMENTARY INFORMATION

		P 1	P 2	P 3	P 4	P 5	P 6	P 7	Avg
**Inter-effectors (M1)**									
superior	L	-17 -33 56	-23 -38 52	-18 -37 54	-17 -33 61	-19 -31 65	-16 -31 66	-18 -33 60	-19 -34 59
	R	16 -36 57	20 -35 51	18 -36 52	17 -27 59	26 -28 59	20 -30 62	23 -28 65	20 -31 58
middle	L	-39 -20 43	-36 -21 39	-39 -23 42	-41 -15 42	-36 -15 49	-38 -15 43	-38 -15 48	-38 -18 44
	R	37 -19 49	35 -23 39	41 -14 44	36 -13 42	39 -13 38	44 -10 48	45 -12 42	40 -15 43
inferior	L	-54 -5 17	-55 -12 11	-55 -2 16	-51 -3 21	-55 3 4	-54 -3 13	-53 -1 14	-54 -3 14
	R	54 -8 16	59 -10 12	51 0 20	62 -1 21	57 4 8	56 1 15	58 4 17	56 -1 16
**Midline (cortex)**									
SMA	L	-6 -10 56	-4 -9 54	-11 -6 53	-4 -8 58	-6 -3 62	-2 -14 64	-9 -8 67	-5 -7 52
	R	11 -2 51	3 -9 46	9 -6 53	3 -6 58	5 -8 68	5 -14 55	6 1 62	5 -5 49
pre-SMA/dACC	L	-5 -5 43	-11 -1 32	-10 4 34	-10 4 41	-6 11 44	-3 -8 49	-8 5 44	-7 1 36
	R	7 -5 37	8 7 28	5 7 36	10 2 46	8 2 52	3 4 46	8 3 43	6 3 36
**Putamen**									
posterior	L	-35 -4 -4	-29 -15 3	-30 -16 3	-25 0 8	-25 -1 -10	-26 -5 13	-25 -5 -6	-28 -5 -1
	R	29 -14 6	29 -14 1	28 -9 1	25 -4 8	30 -10 6	29 -12 10	27 2 -9	28 -9 3
**Thalamus**									
centromedian	L	-9 -23 1	-10 -22 1	-11 -22 1	-9 -20 4	-13 -22 2	-10 -20 3	-10 -20 4	-10 -21 2
	R	11 -22 2	10 -21 1	14 -20 0	9 -19 6	14 -21 4	13 -18 5	12 -18 6	12 -20 3
**Cerebellum**									
dorsal	L	-6 -65 -23	-18 -65 -27	-11 -62 -22	-6 -70 -13	-11 -62 -10	-10 -66 -15	-2 -69 -18	-9 -65 -18
	R	8 -65 -22	23 -62 -26	12 -63 -22	11 -66 -15	7 -60 -5	7 -53 -12	8 -61 -10	11 -61 -16
ventral	L	-21 -57 -65	-19 -58 -65	-28 -55 -63	-24 -50 -47	-28 -45 -47	-23 -47 -50	-16 -57 -44	-23 -53 -54
	R	20 -61 -64	24 -61 -66	26 -57 -62	24 -55 -49	30 -59 -44	24 -54 -47	19 -63 -43	24 -59 -54

Table S1: Inter-effector and connected regions of interest
Locations of inter-effector regions and regions strongly connected to inter-effectors in each highly sampled participant (P1-7), and the average location across participants. Cortical coordinates are centroids of regions; subcortical coordinates are locations of inter-effector functional connectivity peaks within each structure. Coordinates are represented as [X Y Z] in MNI space.

		Avg
**Effector-specific (M1)**		
Foot	L	-6 -14 19
	R	7 -13 19
Hand	L	-14 -8 14
	R	13 -9 15
Mouth	L	-21 -4 10
	R	21 -5 10
**Midline (cortex)**		
CMAd/dACC	L	-5 -1 15
	R	5 -2 14

Table S2: Macaque functional connectivity seed coordinates
Seed coordinates for effector-specific M1 and dorsal cingulate motor area (CMAd) from the group-averaged PRIME-DE macaque data (Extended Data Figure 9; right column). Coordinates are represented as [X Y Z] in MNI space.

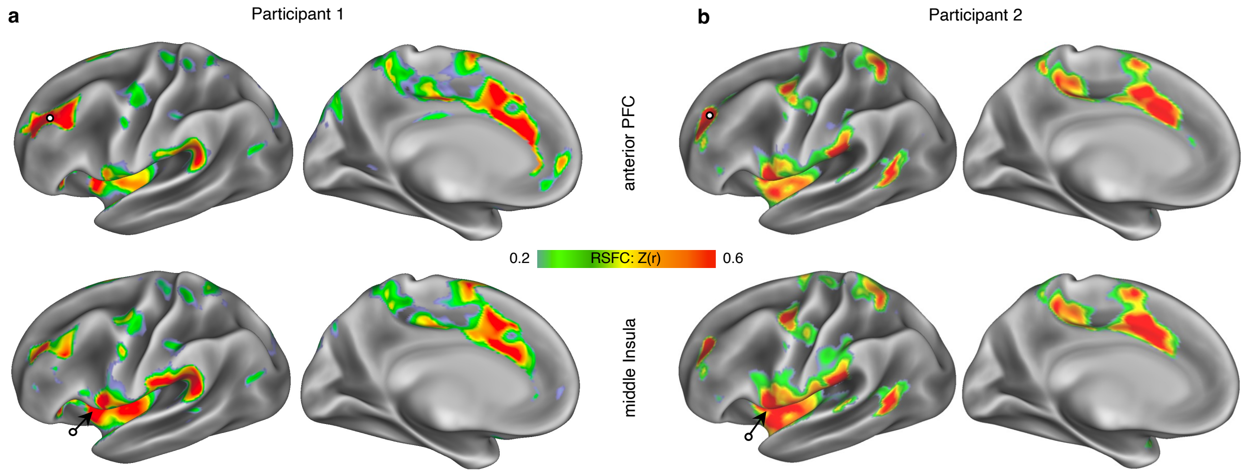

Figure S1| CON regions connected to the inter-effector network. In **a,** Participant 1 and **b,** Participant 2, functional connectivity seeded from the anterior prefrontal cortex (top) and from the middle insula (seed is located under the fold of the inferior frontal gyrus; see arrow). In each case, inter-effector regions exhibited functional connectivity with the seed.

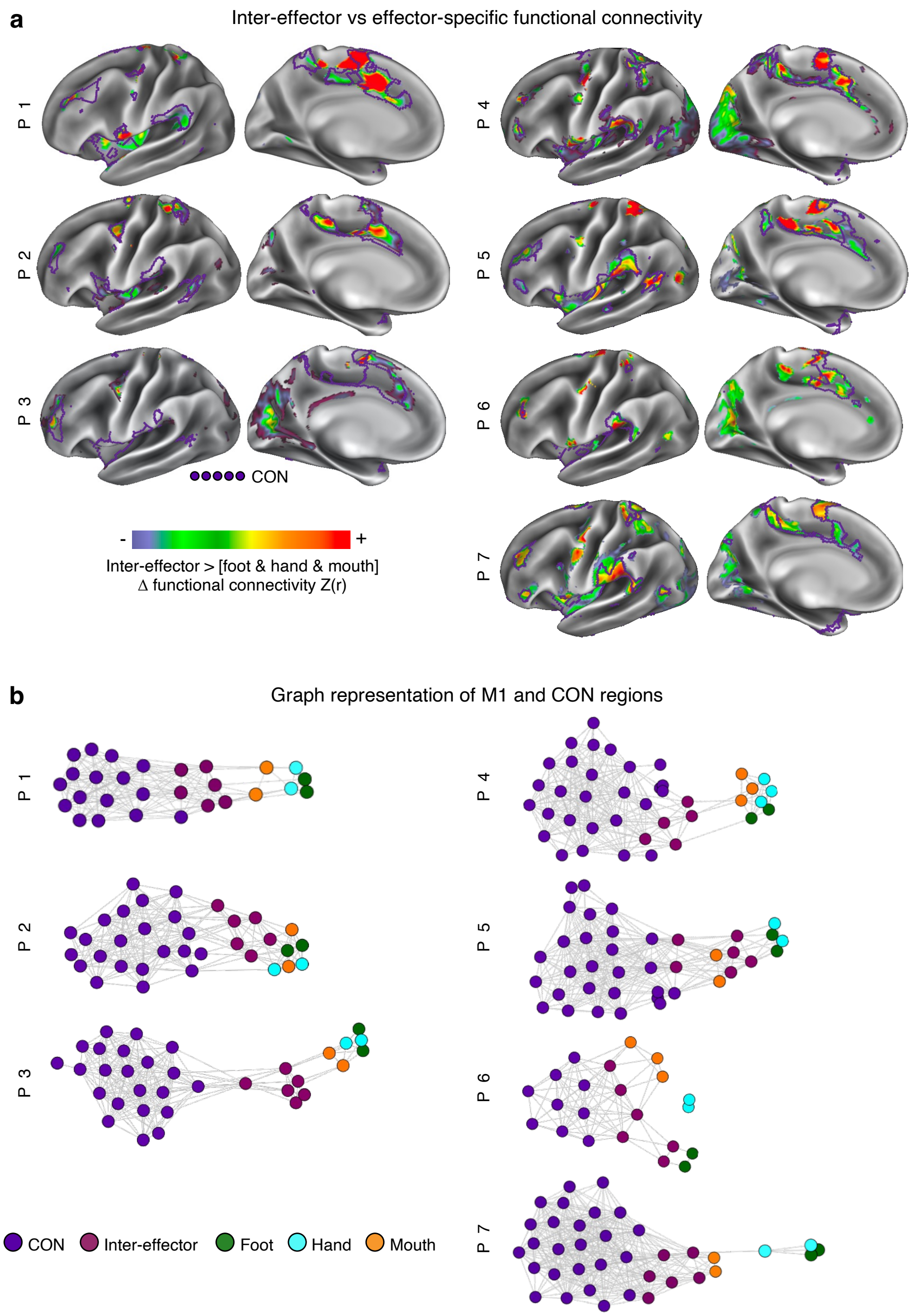

Fig. S2| Inter-effector regions are strongly connected to CON across subjects. a, To quantify functional connectivity differences between the foot/hand/mouth effector and inter-effector regions, we created individual-specific connectivity maps seeded from each of the foot/hand/mouth/inter-effector regions. In every subject, we mapped brain regions more strongly functionally connected to the inter-effector motif than any of the foot/hand/mouth regions. The purple outlines show the individual-specific Cingulo-Opercular Network (CON). Central sulcus regions are masked as they exhibit large differences by definition. **b,** For every participant, relationships between CON, inter-effector, and effector-specific regions are visualized in network space using a spring-embedding plot, in which network nodes are positioned in a 2D plane and connected regions are pulled together while disconnected regions are pushed apart. Connecting lines indicate a strong functional connection.

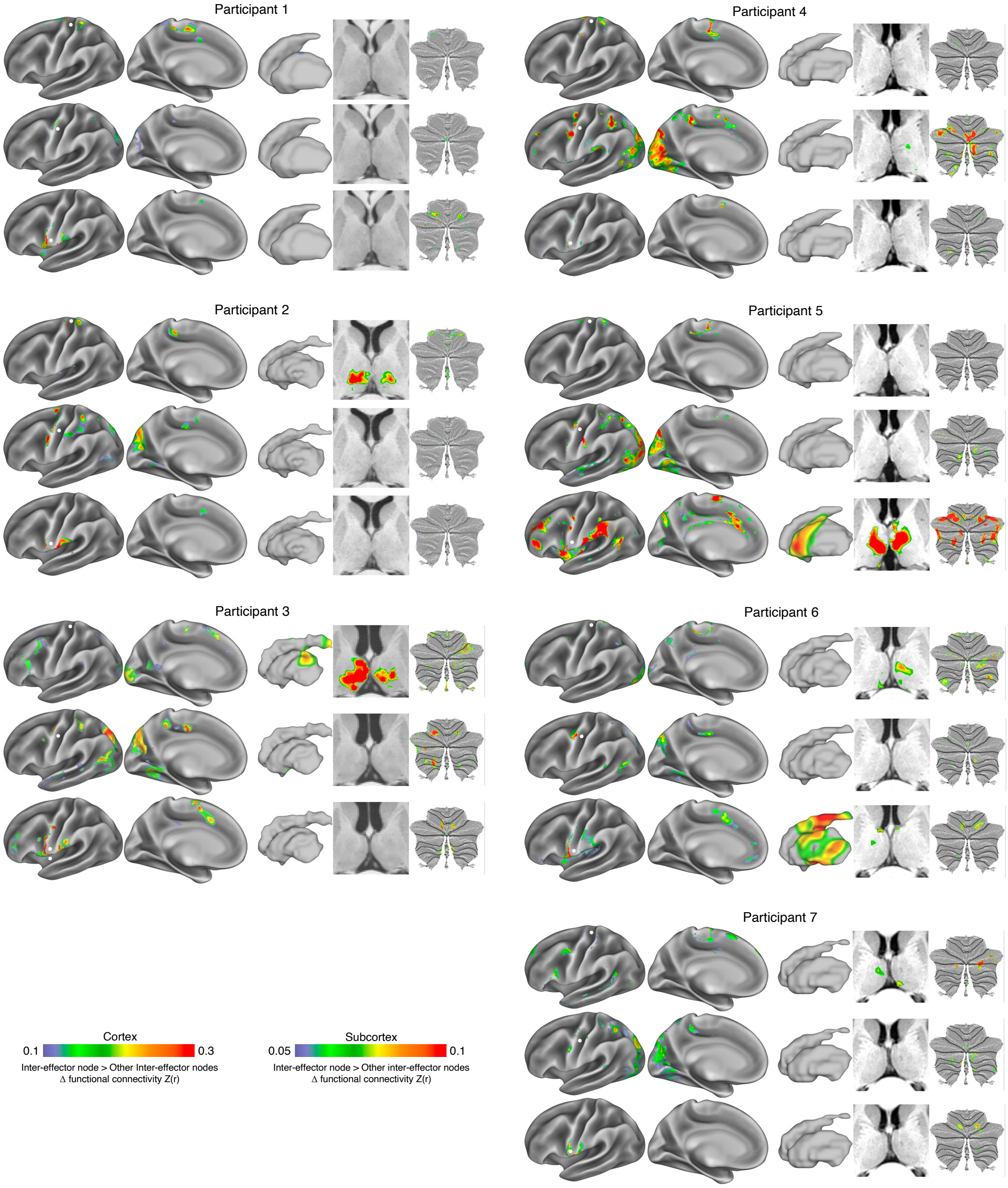

Fig. S3| Functional connectivity differences among inter-effector regions in individual participants. In seven individuals, brain regions more strongly connected to the superior inter-effector region (top row), middle inter-effector region (middle row), and inferior inter-effector region (bottom row) than to either of the other two, in cortex (left), striatum, thalamus, and cerebellum (right). Thresholds used are the same as in Fig. 2b. Note that central sulcus regions are masked as they exhibit large differences by definition. See Extended Data Fig. 5 for overlap across individuals.

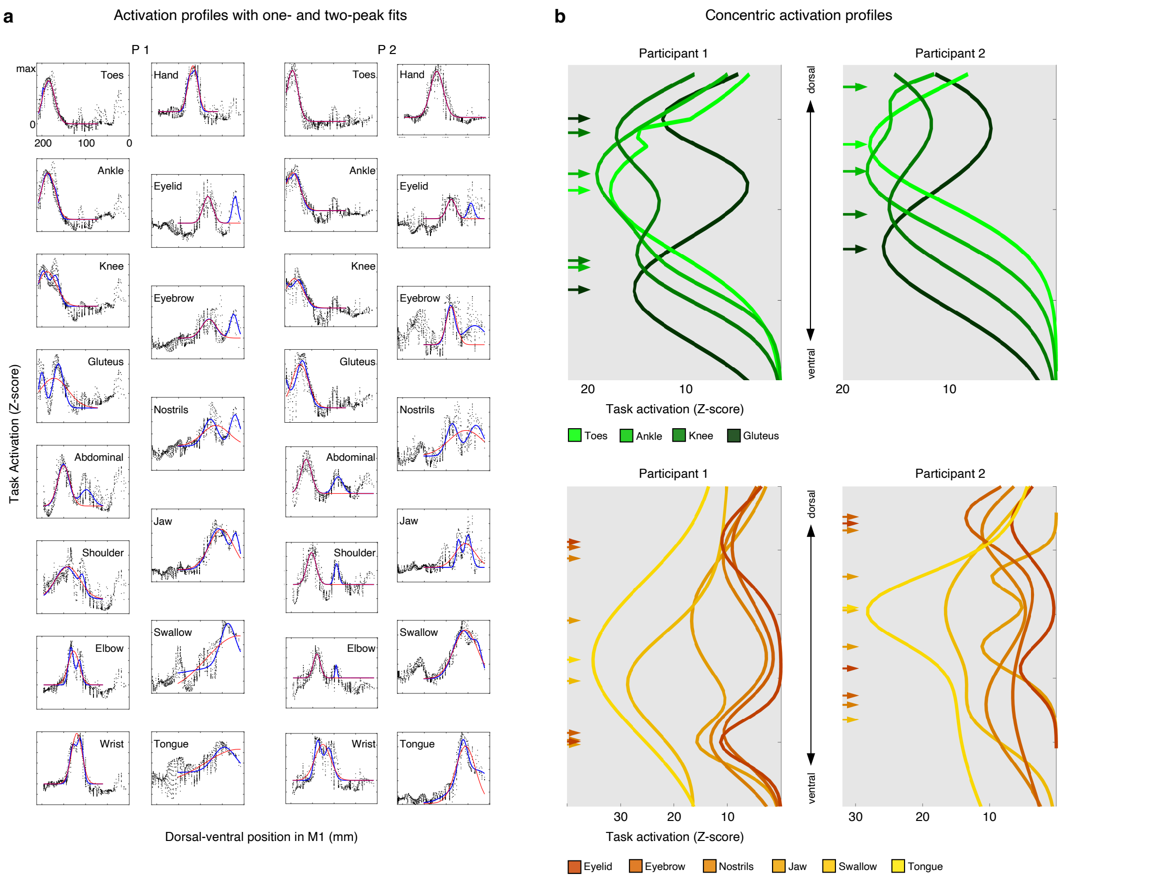

Fig. S4| Concentric organization of task fMRI activations. a, Movement-related activation plotted against dorsal-ventral position within left hemisphere M1 for all movement tasks. For most movements, a two-peak curve (blue), modeled as a double-gaussian, fit the data better than a one-peak curve (red), modeled as a single-gaussian (F-test for comparing models (see methods): $P < 0.001$, FDR corrected). **b,** Fitted two-peak curves are shown for movement of toes, ankle, knee, and gluteus (top), as well as for eyelid, eyebrow, nostrils, jaw, swallow, and tongue (bottom). Activation peaks (arrows on left) are arranged concentrically around the toes (top; green) and tongue (bottom; yellow) peaks.

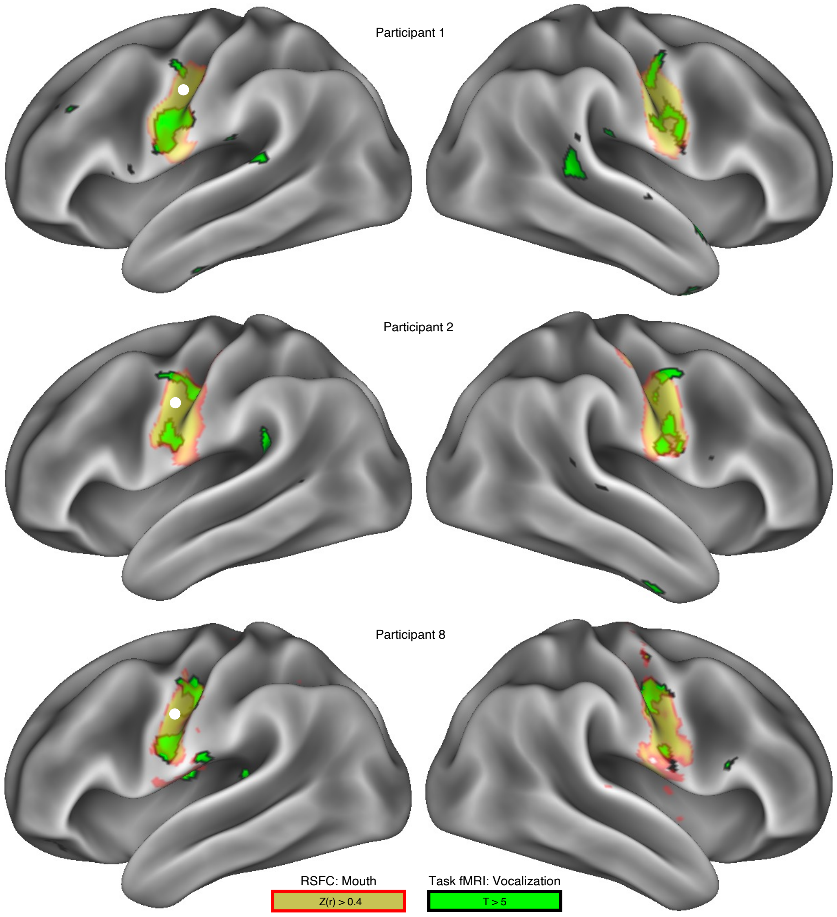

Fig. S5| Vocalization task activation is restricted to the mouth effector region. In three participants, a Voice > [Foot + Hand + Tongue] task fMRI contrast identified vocalization-specific activation in two mirrored regions in precentral gyrus (green). Connectivity seeds placed in the middle of the mouth area revealed that the vocalization-related regions were located within the mouth area, as defined by functional connectivity (red-orange).

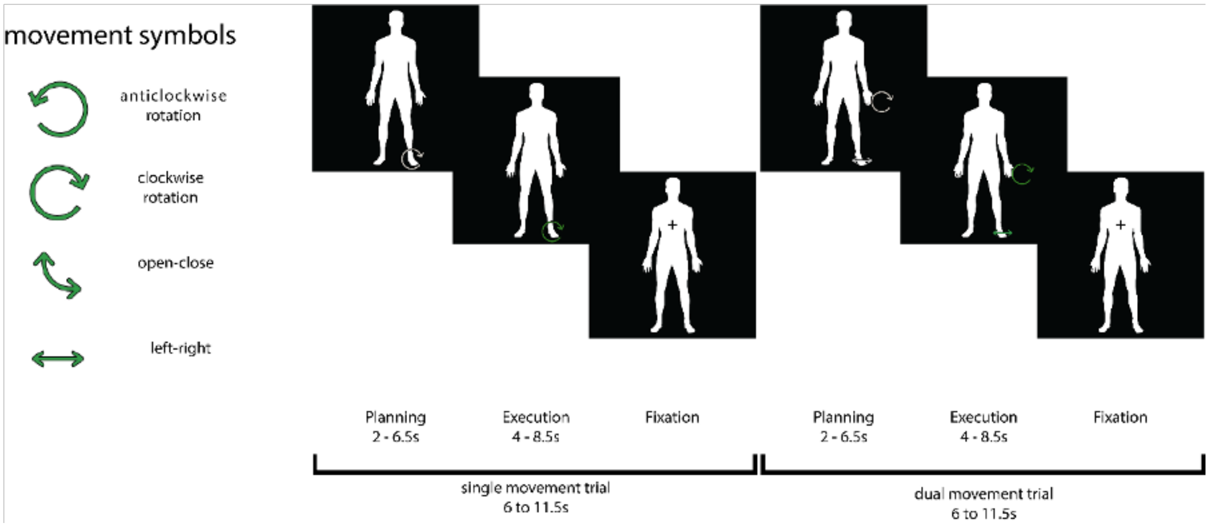

Fig. S6| Illustration of action control task paradigm. Within each run, the participant is prompted to move either a single limb or to simultaneously move two limbs. There are four possible motions— open-close of fingers or toes, left-right movement of the wrist/ankle, clockwise rotation of the wrist/ankle, and counterclockwise rotation of the wrist/ankle—each of which may be executed by any of the four extremities (left or right upper/lower extremity). Each motion/extremity combination may be required in isolation, or in combination with a second simultaneous motion. The participant is cued to prepare the movement(s) when they see one or two movement symbols placed on a body shape in a grey color (planning phase). The participant is instructed to only execute the movement(s) when the grey symbol(s) turn green (execution phase).

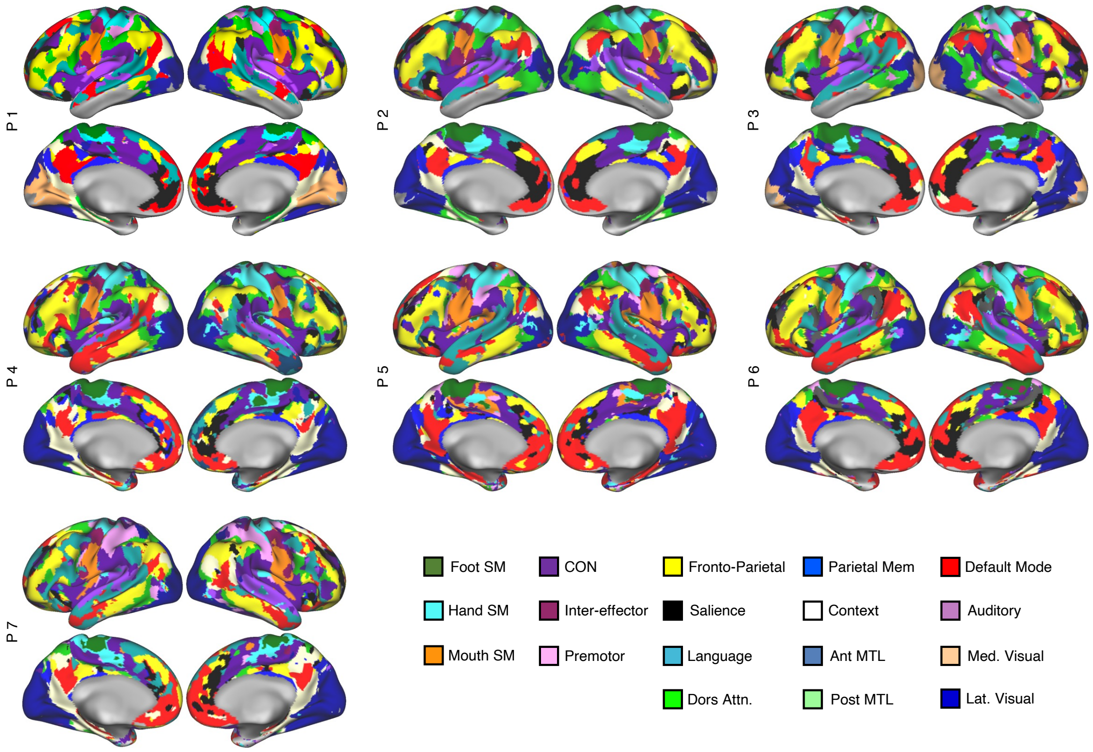

Fig. S7| Individual-specific large-scale brain networks for repeatedly sampled adult participants. To identify classic large-scale networks in each participant, we ran the Infomap algorithm on matrices thresholded at a series of denser thresholds (ranging from 0.1% to 5%), and identified individual-specific networks corresponding to Somatomotor, Inter-effector, Default, Medial and Lateral Visual, Cingulo-Opercular, Fronto-Parietal, Dorsal Attention, Language, Salience, Parietal Memory, and Contextual Association networks, following procedures described in [1].

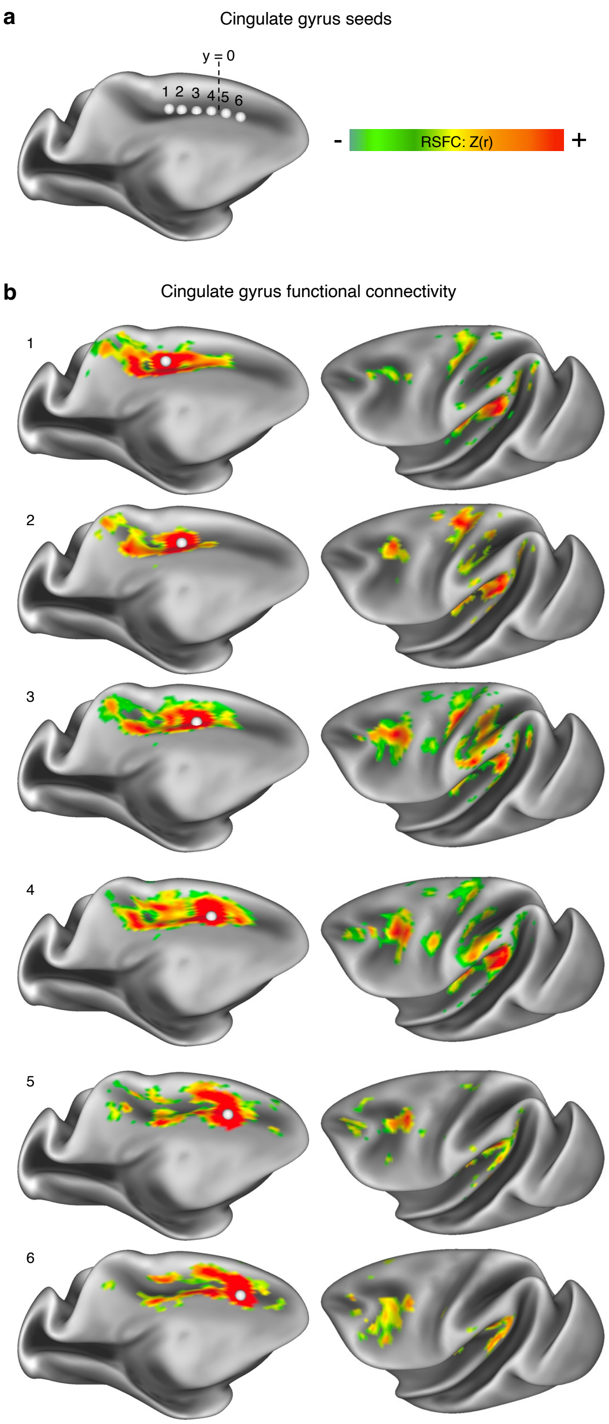

Fig. S8| Functional connectivity in the cingulate gyrus of the macaque. a, Six seeds were placed along the medial prefrontal cortex in posterior dorsal cingulate motor area (CMAd) and rostral cingulate motor area (CMAr). Dotted line denotes the anterior-posterior level of the genu of the arcuate sulcus, taken to be the dividing line between CMAd and CMAr (here at y = 0). **b,** Functional connectivity from each seed in group-averaged macaque data. Distributed connectivity within M1 could be observed seeded from Seed 4 in anterior CMAd.

Supplemental Video Legends

Supplementary Video 1| Complete mapping of functional connectivity in primary motor cortex. Whole-brain functional connectivity is shown seeded from a continuous straight line of points between the dorsomedial and the ventrolateral regions of primary motor cortex in Participant 1.

Supplementary Video 2| Complete mapping of functional connectivity in primary motor cortex across individuals and group-averaged datasets. Whole-brain functional connectivity is shown seeded from a continuous straight line of points between the dorsomedial and the ventrolateral regions of primary motor cortex in **a,** all individuals in the Wash U (left) and Cornell (right) datasets, and **b** in the group-averaged datasets.

Supplemental Discussion

Implications of a mind-body integration system for understanding and treating neurological disorders

The Somato-Cognitive Action Network (SCAN) includes two important thalamic nuclei that serve as deep brain stimulation (DBS) targets for clinical pathologies: the VIM for tremor (e.g., Essential Tremor[2], Parkinson's Disease[2,3]) and the CM for generalized seizures (e.g. Lennox-Gastaut Syndrome[4]). The mechanisms of action underlying these clinical effects are still debated, but it has been proposed that the anti-tremor effects of the VIM may be mediated by its connectivity to the cerebellum, and that the CM's role in arousal may explain its anti-epileptic effects.

Links with the SCAN may be critical for the effects of neuromodulation on movement disorders. Tremor and arousal represent important aspects of integrative action control. Physiological tremor (~ 10 Hz) is thought to time and coordinate movements[5], while physiological arousal and CON engagement are prominent as goal-directed activity begins[6].

Many types of tremors are intention- or goal-related. For example, essential tremor is absent or minimal at rest and is brought out by intentional movements. Further, most tremors disappear in sleep[7]. Finally, tremor and generalized seizures are global phenomena, not characterized by somatotopic specificity. Thus, the effects of VIM and CM DBS are consistent with the modulation of different aspects of whole-body action control, movement timing, and arousal.

Parkinson's disease (PD) may be most specifically related to dysfunction of SCAN circuitry. PD symptoms cut across motor, physiological and volitional domains (e.g., postural instability, autonomic dysfunction, and reduced self-initiated activity, among many others[8]), mirroring SCAN connections to regions relevant for postural control (cerebellar vermis), volition (dACC), and physiological regulation (insula)[9–12]. Work by Clinton Woolsey documented that direct stimulation in M1 of a PD patient temporarily eliminated his whole-body tremor and rigidity[13]. This effect is difficult to explain as a result of stimulation of effector-specific regions but is fairly straightforward as a consequence of SCAN stimulation. Neuronal death in the substantia nigra (SN) is one of the pathophysiological hallmarks of PD. Interestingly, the main target of SN projections is the dorsolateral putamen, which forms part of the Somato-Cognitive Action Network. Inter-effector regions are also strongly functionally connected to the cerebellar vermis, important for postural control and known to be structurally connected to M1 in NHP[14]. In addition, cortical projections important for coordinating physiology with action plans (i.e., blood pressure, orthostasis) primarily originate in CON and anterior M1[11,12]. Thus, if PD is indeed a network disease[15], a fitting candidate for the network most affected by the resulting degeneration is the SCAN.

Finally, the SCAN may be a key locus for the effects of neurostimulation on treatment of chronic pain. Processing of pain involves centromedian thalamic[16], insular, and dorsomedial prefrontal regions, yet chronic pain can in some cases be alleviated by noninvasive stimulation of primary motor cortex[17]. SCAN is strongly connected to all these regions, suggesting that such alleviation of pain symptoms may be enabled by downstream influences of SCAN stimulation.

SUPPLEMENTAL REFERENCES

1. Gordon, E. M. *et al.* Precision Functional Mapping of Individual Human Brains. *Neuron* **95**, 791–807 (2017).
2. Ondo, W., Jankovic, J., Schwartz, K., Almaguer, M. & Simpson, R. K. Unilateral thalamic deep brain stimulation for refractory essential tremor and Parkinson's disease tremor. *Neurology* **51**, 1063–1069 (1998).
3. Fasano, A., Daniele, A. & Albanese, A. Treatment of motor and non-motor features of Parkinson's disease with deep brain stimulation. *The Lancet Neurology* **11**, 429–442 (2012).
4. Valentín, A. *et al.* Deep brain stimulation of the centromedian thalamic nucleus for the treatment of generalized and frontal epilepsies. *Epilepsia* **54**, 1823–1833 (2013).
5. Llinás, R. R. *I of the vortex: From neurons to self*. x, 302 (The MIT Press, 2001).
6. Dosenbach, N. U. F., Fair, D. A., Cohen, A. L., Schlaggar, B. L. & Petersen, S. E. A dual-networks architecture of top-down control. *Trends in Cognitive Sciences* **12**, 99–105 (2008).
7. Kakei, S., Manto, M., Tanaka, H. & Mitoma, H. Pathophysiology of Cerebellar Tremor: The Forward Model-Related Tremor and the Inferior Olive Oscillation-Related Tremor. *Frontiers in Neurology* **12**, (2021).
8. Dauer, W. & Przedborski, S. Parkinson's disease: mechanisms and models. *Neuron* **39**, 889–909 (2003).
9. Siegel, J. S. *et al.* The circuitry of abulia: Insights from functional connectivity MRI. *NeuroImage: Clinical* **6**, 320–326 (2014).
10. Darby, R. R., Joutsa, J., Burke, M. J. & Fox, M. D. Lesion network localization of free will. *PNAS* **115**, 10792–10797 (2018).
11. Wall, P. D. & Davis, G. D. Three cerebral cortical systems affecting autonomic function. *J Neurophysiol* **14**, 507–517 (1951).
12. Pool, J. L. & Ransohoff, J. Autonomic effects on stimulating rostral portion of cingulate gyri in man. *J Neurophysiol* **12**, 385–392 (1949).
13. Woolsey, C. N., Erickson, T. C. & Gilson, W. E. Localization in somatic sensory and motor areas of human cerebral cortex as determined by direct recording of evoked potentials and electrical stimulation. *J Neurosurg* **51**, 476–506 (1979).
14. Coffman, K. A., Dum, R. P. & Strick, P. L. Cerebellar vermis is a target of projections from the motor areas in the cerebral cortex. *Proceedings of the National Academy of Sciences* **108**, 16068–16073 (2011).
15. Braak, H. *et al.* Staging of brain pathology related to sporadic Parkinson's disease. *Neurobiology of Aging* **24**, 197–211 (2003).
16. Weigel, R., Weigel, R., Krauss, J. K. & Krauss, J. K. Center Median-Parafascicular Complex and Pain Control. *SFN* **82**, 115–126 (2004).
17. Jensen, M. P., Day, M. A. & Miró, J. Neuromodulatory treatments for chronic pain: efficacy and mechanisms. *Nat Rev Neurol* **10**, 167–178 (2014).
