# Supplementary material for: A somato-cognitive action network alternates with effector regions in motor cortex
Source: Nature. 2023 Apr 19;617(7960):351–9. doi: 10.1038/s41586-023-05964-2 (PMC10172144; doi:10.1038/s41586-023-05964-2)
Supplement: Supplementary file 2 — Reporting Summary [file 41586_2023_5964_MOESM2_ESM.pdf]

nature portfolio

Corresponding author(s): Evan M. Gordon

Last updated by author(s): Feb 27, 2023

Reporting Summary

Nature Portfolio wishes to improve the reproducibility of the work that we publish. This form provides structure for consistency and transparency in reporting. For further information on Nature Portfolio policies, see our Editorial Policies and the Editorial Policy Checklist.

Statistics

For all statistical analyses, confirm that the following items are present in the figure legend, table legend, main text, or Methods section.

n/a	Confirmed	
☐	☒	The exact sample size (*n*) for each experimental group/condition, given as a discrete number and unit of measurement
☐	☒	A statement on whether measurements were taken from distinct samples or whether the same sample was measured repeatedly
☐	☒	The statistical test(s) used AND whether they are one- or two-sided<br>*Only common tests should be described solely by name; describe more complex techniques in the Methods section.*
☒	☐	A description of all covariates tested
☐	☒	A description of any assumptions or corrections, such as tests of normality and adjustment for multiple comparisons
☐	☒	A full description of the statistical parameters including central tendency (e.g. means) or other basic estimates (e.g. regression coefficient) AND variation (e.g. standard deviation) or associated estimates of uncertainty (e.g. confidence intervals)
☐	☒	For null hypothesis testing, the test statistic (e.g. *F*, *t*, *r*) with confidence intervals, effect sizes, degrees of freedom and *P* value noted<br>*Give P values as exact values whenever suitable.*
☒	☐	For Bayesian analysis, information on the choice of priors and Markov chain Monte Carlo settings
☒	☐	For hierarchical and complex designs, identification of the appropriate level for tests and full reporting of outcomes
☐	☒	Estimates of effect sizes (e.g. Cohen's *d*, Pearson's *r*), indicating how they were calculated

Our web collection on statistics for biologists contains articles on many of the points above.

Software and code

Policy information about availability of computer code

Data collection	Task stimuli were presented using JSpsych toolbox v6.3, available from https://www.jspsych.org/6.3/
Data analysis	Data processing code for the ABCD data, as well as for the Child subject, can be found here: https://github.com/DCAN-Labs/abcd-hcp-pipeline<br>Data processing code for the HCP data can be found here: https://github.com/Washington-University/HCPpipelines<br>Data processing code for P01-03, P08, the Neonate, Infant, and Perinatal Stroke subjects can be found here: https://gitlab.com/DosenbachGreene/<br>Data processing code for P04-07 can be found here: https://github.com/cjl2007/Liston-Laboratory-MultiEchofMRI-Pipeline<br>Data processing code for the Macaque datasets can be found here: https://github.com/DCAN-Labs/nhp-abcd-bids-pipeline<br>Code specific to the analyses in this manuscript can be found here: https://gitlab.com/DosenbachGreene/SCAN/<br><br>Software packages incorporated into the above pipelines for data analysis included:<br>Matlab R2020b, https://www.mathworks.com/<br>Connectome Workbench 1.5, http://www.humanconnectome.org/software/connectome-workbench.html<br>Freesurfer v6.2, https://surfer.nmr.mgh.harvard.edu/<br>FSL 6.0, https://fsl.fmrib.ox.ac.uk/fsl/fslwiki<br>4dfp tools, https://4dfp.readthedocs.io/en/latest/<br>Infomap, www.mapequation.org

For manuscripts utilizing custom algorithms or software that are central to the research but not yet described in published literature, software must be made available to editors and reviewers. We strongly encourage code deposition in a community repository (e.g. GitHub). See the Nature Portfolio guidelines for submitting code & software for further information.

Data

Policy information about availability of data

All manuscripts must include a data availability statement. This statement should provide the following information, where applicable:

- Accession codes, unique identifiers, or web links for publicly available datasets
- A description of any restrictions on data availability
- For clinical datasets or third party data, please ensure that the statement adheres to our policy

Data from individual subjects P01-P03 is available in the openneuro repository here: https://openneuro.org/datasets/ds002766/versions/3.0.0

Data from the individual Perinatal Stroke subject is available in the openneuro repository: https://openneuro.org/datasets/ds004498/versions/1.0.0.

Data from the UKB dataset used here is available at https://www.fmrib.ox.ac.uk/ukbiobank/

The ABCD data used in this report came from ABCD collection 3165 and the Annual Release 2.0, DOI 10.15154/1503209.

Data from the HCP dataset used here is available at www.humanconnectome.org. Users must agree to data use terms for the HCP before being allowed access to the data and ConnectomeDB, details are provided at https://www.humanconnectome.org/study/hcp-young-adult/data-use-terms.

Data from the WU120 dataset is available in the openneuro repository here: https://openneuro.org/datasets/ds000243/versions/00001

Data from the PRIME-DE Oxford macaque dataset used in this report are available at https://fcon_1000.projects.nitrc.org/indi/PRIME/oxford.html. Users register with NITRC and with the 1000 Functional Connectomes Project website on NITRC to gain access to the PRIME-DE datasets.

Data from the UMN macaque will be publicly available via the PRIME-DE website (see above) by the end of 2023, after data collection of a larger sample is complete.

Data from individual subjects P04-P07, P08, the individual Neonate, Infant, and Child subjects, as well as from the group average Infant datasets, are available on reasonable request from authors CL, EMG, JRP, CMS, and DJG, and CDS, respectively. They are not yet available through public databases, because data collection is still ongoing.

The DISTAL atlas is available from https://www.lead-dbs.org/helpsupport/knowledge-base/atlasesresources/distal-atlas/
The SUIT atlas is available from https://www.diedrichsenlab.org/imaging/suit.htm

Human research participants

Policy information about studies involving human research participants and Sex and Gender in Research.

Reporting on sex and gender

Findings apply to all studied individuals and groups, regardless of sex.

Sex ratios:
P01-03, P08: 3M 1F
P04-07: 4M
Neonate: 1M
Infant: 1M
Child: 1 M
Perinatal Stroke: 1 M
UKB: 47%M, 53%F
ABCD: 49%M, 51%F
HCP: 402M, 410F
WU120: 60M, 60F
eLABE: 141M, 121F

Population characteristics

Age ranges:

P01-03, P08: 25-40y
P04-07: 24-38y
Neonate: 13 days
eLABE: 1-3 weeks
Infant: 11 months
Child: 9y
Perinatal Stroke: 13y
ABCD: 9-10y
HCP: 22-35
UKB: 40-69
WU120: 19-32y

Recruitment

P01-03, P08, WashU120: Healthy adult participants were recruited from the Washington University community via flyers and word of mouth.

P04-07: Healthy adult participants were recruited from the Weill Cornell Medical School community via word of mouth.

Infant, Child: Parents were recruited from the St. Louis community via flyers and word of mouth.

Perinatal Stroke: the participant was referred to the neurology clinic of author NUFD (St Louis Children's Hospital) because of noted clumsiness of his right hand.

Neonate and group-averaged Neonate: Mothers were recruited during the 2nd or 3rd trimester from two obstetrics clinics at Washington University.

HCP: Sampling 300–400 young adult sibships of average size 3–4, with most of these sibships including a MZ or DZ twin pair.

ABCD: A very important motivation for the ABCD study is that its sample should reflect, as best as possible, the sociodemographic variation of the US population.The ABCD cohort recruitment emulates a multi-stage probability sample of eligible children: A nationally distributed set of 21 primary stage study sites, a probability sampling of schools within the defined catchment areas for each site, and recruitment of eligible children in each sample school. The major departure from traditional probability sampling of U.S. children originates in how participating neuroimaging sites were chosen for the study. Although the 21 ABCD study sites are well-distributed nationally the selection of collaborating sites is not a true probability sample of primary sampling units (PSUs) but was constrained by the grant review selection process and the requirement that selected locations have both the research expertise and the neuroimaging equipment needed for the study protocol.

UKB: Participants were assessed between 2006 and 2010 in 22 assessment centres throughout the UK, covering a variety of different settings to provide socioeconomic and ethnic heterogeneity and urban–rural mix. This ensured a broad distribution across all exposures to allow the reliable detection of generalisable associations between baseline characteristics and health outcomes.

Summary: While each individual dataset represented here may contain selection biases, particularly including socio-demographic factors, the replication of findings across all datasets provides confidence that the findings do not depend on these factors.

Ethics oversight

P01-03, P08, Neonate, Infant, Child, Perinatal Stroke, HCP, WU120, and group average Neonate datasets: The study was approved by the Washington University School of Medicine Human Studies Committee and Institutional Review Board.

P04-07: The study was approved by the Weill Cornell Medicine Institutional Review Board.

ABCD: The ABCD Study obtained centralized institutional review board approval from the University of California, San Diego, and each of the 21 study sites obtained local institutional review board approval.

UKB: Ethical procedures are controlled by a dedicated Ethics and Guidance Council (http://www.ukbiobank.ac.uk/ethics) that has developed with UK Biobank an Ethics and Governance Framework (given in full at http://www.ukbiobank.ac.uk/wp-content/uploads/2011/05/EGF20082.pdf), with IRB approval also obtained from the North West Multi-center Research Ethics Committee.

Note that full information on the approval of the study protocol must also be provided in the manuscript.

Field-specific reporting

Please select the one below that is the best fit for your research. If you are not sure, read the appropriate sections before making your selection.

☒ Life sciences ☐ Behavioural & social sciences ☐ Ecological, evolutionary & environmental sciences

For a reference copy of the document with all sections, see nature.com/documents/nr-reporting-summary-flat.pdf

Life sciences study design

All studies must disclose on these points even when the disclosure is negative.

Sample size

Much of this study was focused on within- rather than across-individual analysis. In these analyses, the relevant factor is ensuring that enough data is collected from each participant to ensure reliable measures. We have previously shown that this requires a minimum of 30 minutes of data per individual, and that reliability continuously improves as more per-individual data is collected (Laumann et al., 2015; Gordon et al., 2017). Therefore we ensured that all participants tested at the individual level had at least that much data, and that in many cases the data quantities were much higher.

For analyses of group-averaged data, we always employed the maximum number of participants available in public datasets.

Data exclusions

ABCD: 3,928 participants were selected as the participants with at least 8 minutes of low-motion data, a pre-established criterion.

HCP: 812 participants were selected who completed four 15-minute resting-state fMRI runs and who had their raw data reconstructed using the newer "recon 2" software, a pre-established criterion.

PRIME-DE: Each animal's data was closely visually inspected for quality. Following these inspections, data from eleven animals were excluded

due to the presence of artifact in or near the central sulcus, leaving eight animals in the final data. This criterion was not pre-established but was necessary given the observation of artifact in some macaques.

Replication	Experimental findings were replicated in independent data collected from all human individuals (aside from the neonate data) and group-averaged datasets, as well as within-participant (i.e. by repeats) in P01-03.
Randomization	N/A: There were no separate experimental groups.
Blinding	N/A: There were no separate experimental groups.

Reporting for specific materials, systems and methods

We require information from authors about some types of materials, experimental systems and methods used in many studies. Here, indicate whether each material, system or method listed is relevant to your study. If you are not sure if a list item applies to your research, read the appropriate section before selecting a response.

Materials & experimental systems

n/a	Involved in the study
☒	☐ Antibodies
☒	☐ Eukaryotic cell lines
☒	☐ Palaeontology and archaeology
☐	☒ Animals and other organisms
☒	☐ Clinical data
☒	☐ Dual use research of concern

Methods

n/a	Involved in the study
☒	☐ ChIP-seq
☒	☐ Flow cytometry
☐	☒ MRI-based neuroimaging

Animals and other research organisms

Policy information about studies involving animals; ARRIVE guidelines recommended for reporting animal research, and Sex and Gender in Research

Laboratory animals	The study included one adult female macaca fascicularis (age 6 y) and eight adult male macaca mulatta (age 4.1 +/- 0.98 years)
Wild animals	The study did not include wild animals
Reporting on sex	The UMN macaque was female and the PRIME-DE macaques were male. Sex was not considered as a factor here because it was confounded with site.
Field-collected samples	The study did not include samples collected from the field
Ethics oversight	UMN macaque: Experimental procedures were carried out in accordance with the University of Minnesota Institutional Animal Care and Use Committee and the National Institute of Health standards for the care and use of non-human primates.<br><br>PRIME-DE macaques: Protocols for animal care, magnetic resonance imaging, and anaesthesia were carried out under authority of personal and project licenses in accordance with the UK Animals (Scientific Procedures) Act 1986.

Note that full information on the approval of the study protocol must also be provided in the manuscript.

Magnetic resonance imaging

Experimental design

Design type	Resting state, block design task, and event-related task
Design specifications	Resting state: 172 – 1,813 total minutes of data acquired per participant<br><br>Block design movement task battery: 10 15.4-second movement blocks across five conditions plus 3 15.4-second rest blocks per run; 64 runs per subject.<br><br>Block design laryngeal mapping task: 12 15.0-second movement blocks across six conditions plus 2 15.0-second rest blocks per run; 10 runs per subject.<br><br>Even related action control task: . The participant is cued to prepare the movement(s) when they see one or two movement symbols placed on a body shape in a grey color (planning phase), and is then cued to execute the movement(s) when the grey symbol or symbols turn green (execution phase). Using a pseudorandom jitter, the planning phase can last from 2 to 6.5s followed by 4 to 8.5s of movement execution. Each movement trial (planning and

execution) is followed by a jittered fixation of up to 5s. A rest block of 8.6s is implemented every 12 movements. 48 trials were collected in each run; twelve runs were acquired in each participant.

Behavioral performance measures	Behavioral outputs were not recorded.

Acquisition

Imaging type(s)	Structural (T1-w and T2w), Diffusion, Functional
Field strength	3.0T

Sequence & imaging parameters

P01, P03, P08: A high-resolution T1-weighted MP-RAGE (TE=2.22ms, TR=2400ms, flip angle=8°, 208 slices with 0.8x0.8x0.8mm voxels) and a T2-weighted spin-echo image (TE=563ms, TR=3200ms, flip angle=120°, 208 slices with 0.8x0.8x0.8mm voxels) were collected.

P02: four T1-weighted images (sagittal, 224 slices, 0.8 mm isotropic resolution, TE=3.74 ms, TR=2400 ms, TI=1000 ms, flip angle = 8°) and four T2-weighted images (sagittal, 224 slices, 0.8 mm isotropic resolution, TE=479 ms, TR=3200 ms).
P01-03: fMRI scans were collected as a blood oxygen level-dependent (BOLD) contrast sensitive gradient echo-planar sequence (TE=33ms, flip angle=84°, resolution=2.6 mm isotropic, TR=1100ms, multiband 4 acceleration). Diffusion imaging was collected using a single-shot echo planar diffusion-weighted sequence consisting of 75 contiguous axial slices, isotropic (2x2x2 mm^3) resolution, TR/TE 3500/83 ms, four shells (b-values 0.25, 0.5, 1.0, and 1.5 ms/mm2) and 96 encoding directions.

P01, P02 (laryngeal mapping fMRI task), P08 (all fMRI): fMRI scans were collected as a multi-band five-echo blood oxygen level-dependent (BOLD) contrast sensitive gradient echo-planar sequence (flip angle = 68°, resolution = 2.0 mm isotropic, TR = 1761ms, multiband 6 acceleration, TE1: 14.20 ms, TE2: 38.93 ms, TE3: 63.66 ms, TE4: 88.39 ms, and TE5: 113.12 ms)

P04-07: High-resolution T1-weighted MP-RAGE images (TE=2.28ms, TR=2400ms, flip angle=90°, 208 slices with 0.8x0.8x0.8mm voxels) and T2-weighted spin-echo images (TE=563ms, TR=3200ms, flip angle=8°, 208 slices with 0.8x0.8x0.8mm voxels) were acquired. A 14.5 minute long multi-echo resting-state fMRI scan was collected as a five-echo blood oxygen level-dependent (BOLD) contrast sensitive gradient echo-planar sequence (flip angle=68°, resolution=2.4 mm isotropic, TR=1355ms, multiband 6 acceleration, TE1: 13.40 ms, TE2: 31.11 ms, TE3: 48.82 ms, TE4: 66.53 ms, and TE5: 84.24 ms).

Neonate: A high-resolution T2-weighted spin-echo image was collected (TE=563ms, TR=3200ms, flip angle=120°, 208 slices with 0.8x0.8x0.8mm voxels). Multi-echo resting-state fMRI runs were collected as a five-echo blood oxygen level-dependent (BOLD) contrast sensitive gradient echo-planar sequence (flip angle=68°, resolution=2.0 mm isotropic, TR=1761ms, multiband 6 acceleration, TE1: 14.20 ms, TE2: 38.93 ms, TE3: 63.66 ms, TE4: 88.39 ms, and TE5: 113.12 ms).

Infant: a high-resolution T1-weighted MP-RAGE (TE=2.24ms, TR=2400ms, flip angle=8°, 208 slices with 0.8x0.8x0.8mm voxels) and a T2-weighted spin-echo image (TE=564ms, TR=3200ms, flip angle=120°, 208 slices with 0.8x0.8x0.8mm voxels) were collected. Resting-state fMRI was collected as a blood oxygen level-dependent (BOLD) contrast sensitive gradient echo-planar sequence (flip angle=52°, resolution=3.0 mm isotropic, TE=30ms, TR=861ms, multiband 4 acceleration).

Child: High-resolution T1-weighted MP-RAGE images (TE=2.90ms, TR=2500ms, flip angle=8°, 176 slices with 1mm isotropic voxels), and T2-weighted spin-echo images (TE=564ms, TR=3200ms, flip angle=120°, 176 slices with 1mm isotropic voxels) were collected. Resting-state fMRI was collected as a blood oxygen level-dependent (BOLD) contrast sensitive gradient echo-planar sequence (flip angle=84°, resolution=2.6mm isotropic, 56 slices, TE=33ms, TR=1100ms, multiband 4 acceleration).

Perinatal stroke: T1-weighted images (sagittal, 224 slices, 0.8 mm isotropic resolution, TE=3.74 ms, TR=2400 ms, TI=1000 ms, flip angle = 8 degrees), and T2-weighted images (sagittal, 224 slices, 0.8 mm isotropic resolution, TE=479 ms, TR=3200 ms) were collected. Resting state fMRI data were collected using a gradient-echo EPI sequence (TR = 2.2 s, TE = 27 ms, flip angle = 90°, voxel size = 4 mm x 4 mm x 4 mm, 36 slices).

UMN macaque: A T1 weighted MP-RAGE was acquired (TR = 3300 ms, TE = 3.56 ms, TI = 1140, flip angle = 5°, slices = 256, matrix = 320×260, acquisition voxel size = 0.5 × 0.5 × 0.5 mm 3, in-plane acceleration GRAPPA = 2). A resolution and FOV matched T2 weighted 3D turbo spin-echo sequence was also acquired. FMRI timeseries, each consisting of 700 continuous 2D multiband EPI89–91 functional volumes (TR = 1110ms; TE = 17.6 ms; flip angle = 60°, slices = 58, matrix = 108×154; FOV = 81×115.5 mm ; acquisition voxel size = 0.75 × 0.75 × 0.75 mm) were acquired with a left-right phase encoding direction using in plane acceleration factor GRAPPA = 3, partial Fourier = 7/8th, and MB factor = 2.

PRIME-DE macaques: A T1-weighted MPRAGE sequence was used to acquire anatomical data (TR = 2500 ms, TE = 4.01 ms, TI = 1100, flip angle = 8°, acquisition voxel size = 0.5 × 0.5 × 0.5 mm, 128 slices). Festing-state fMRI data was acquired at a 2.0 mm isotropic voxel resolution (TR = 2000 ms, TE = 19 ms, Flip angle = 90°).

UKB: T1 - 1.0x1.0x1.0 mm, 208x256x256 3D MPRAGE, sagittal, R=2, TI/TR=880/2000 ms; T2 FLAIR - 1.05x1.0x1.0 mm 192x256x256 FLAIR, 3D SPACE, sagittal, R=2, PF 7/8, fat sat, TI/TR=1800/5000 ms, elliptical; resting-state fMRI - 2.4x2.4x2.4 mm, 88x88x64 TE/TR=39/735 ms, MB=8, R=1, flip angle 52°, fat sat.

HCP: T1w images are acquired using a 3D MPRAGE sequence with 0.7 mm isotropic resolution (FOV = 224 mm, matrix = 320, 256 sagittal slices in a single slab), TR = 2400 ms, TE = 2.14 ms, TI = 1000 ms, FA = 8°, Bandwidth (BW) = 210 Hz per

pixel, Echo Spacing (ES) = 7.6 ms, with a non-selective binomial (1:1) water excitation pulse (a pair of 100 µs hard pulses with 1.2 ms spacing) to reduce signal from bone marrow and scalp fat, phase encoding undersampling factor GRAPPA = 2. T2w images are acquired using the variable flip angle turbo spin-echo sequence (Siemens SPACE;) with 0.7 mm isotropic resolution (same matrix, FOV, and slices as in the T1w), TR = 3200 ms, TE = 565 ms, BW = 744 Hz per pixel, no fat suppression pulse, phase encoding undersampling factor GRAPPA = 2, total turbo factor = 314 (to be achieved with a combination of turbo factor and slice turbo factor, when available), echo train length of 1105 echoes. Resting-state fMRI gradient echo EPI images are 2 mm isotropic resolution (FOV: 208 mm × 180 mm, Matrix: 104 × 90 with 72 slices covering the entire brain), acquired as pairs of R->L and L->R phase encoding directions.

ABCD: T1w images - 3D MPRAGE, 256x256x176, 1.0mm isotropic, TR=2500ms, TE=2.88ms, TI=1060ms, Flip angle=8°, R=2. T2w images - variable flip angle turbo spin-echo, 256x256x176, 1.0mm isotropic, TR=3200ms, TE=565ms, R=2. Resting-state fMRI - gradient echo EPI images, 90x90 with 60 slices, 2.4mm isotropic, TR=800ms, TE=30ms, flip angle = 52°, Multiband factor=6.

WashU120: T1-weighted images (TE=3.08 ms, TR(partition)=2.4 s, TI=1000 ms, flip angle=8°, 176 slices with 1×1×1 mm voxels). A T2-weighted turbo spin echo structural image (TE=84 ms, TR=6.8 s, 32 slices with 1×1×4 mm voxels) was acquired. Resting-state fMRI was performed using a blood oxygenation level-dependent (BOLD) contrast sensitive gradient echo echo-planar sequence (TE=27 ms, flip angle=90°, in-plane resolution=4×4 mm). Whole brain EPI volumes (MR frames) of 32 contiguous, 4 mm-thick axial slices were obtained every 2.5 seconds.

Area of acquisition

Whole-brain

Diffusion MRI ☒ Used ☐ Not used

Parameters

Diffusion imaging was collected using a single-shot echo planar diffusion-weighted sequence consisting of 75 contiguous axial slices, isotropic (2x2x2 mm^3) resolution, TR/TE 3500/83 ms, four shells (b-values 0.25, 0.5, 1.0, and 1.5 ms/mm2) and 96 encoding directions.

Preprocessing

Preprocessing software

FSL 6.0 software tools used: FAST, Eddy, Topup, DTIFit, FEAT
Freesurfer versions 5.0, 5.3, and 6.0, recon-all pipelines for brain segmentation
Connectome workbench v1.0 and 1.5
4dfp tools (https://4dfp.readthedocs.io/)
Processing pipelines used:
https://github.com/DCAN-Labs/abcd-hcp-pipelines
https://github.com/DCAN-Labs/nhp-abcd-bids-pipeline
Smoothing kernels employed: from 2mm to 6mm FWHM in humans and 1.5mm in macaques (geodesic on cortical surface)

Normalization

P01-03, P08, Infant, Perinatal Stroke: BOLD->T2 rigid body linear, T2->T1 rigid body linear, T1-> atlas linear
WU120: BOLD->T1 rigid body linear, T1-> atlas linear
Neonate: BOLD->T2 rigid body linear, T2-> atlas linear
All other datasets: BOLD->T2 rigid body linear, T2->T1 rigid body linear, T1-> atlas nonlinear

Normalization template

P01-03, Perinatal stroke, and WU120: Talaraich
All other human datasets: MNI
Macaque dataset: Yerkes 19

Noise and artifact removal

P01-03, P08, Neonate, group Neonate, Infant, Perinatal Stroke:
Denoising of resting-state fMRI data was accomplished by regression of nuisance waveforms following a CompCor-like strategy. Regressors included the 6 rigid parameters derived by retrospective motion correction, the global signal averaged over the brain, and orthogonalized waveforms extracted from the ventricles, white matter and extra-cranial tissues (excluding the eyes). Frame censoring (scrubbing) was computed on the basis of both frame-wise displacement (FD) and variance of derivatives (DVARS) measures with thresholds set individually for each participant. Gray plot displays were visually checked to confirm artifact reduction. The data then were temporally band-pass filtered prior to nuisance regression, retaining frequencies between 0.005 Hz and 0.1 Hz. Censored frames were replaced by linearly interpolated values prior to filtering.

P04-07: Multi-echo ICA (ME-ICA) denoising designed to isolate spatially structured T2*- (neurobiological; “BOLD-like”) and S0-dependent (non-neurobiological; “not BOLD-like”) signals was performed using a modified version of the “tedana.py” workflow (https://tedana.readthedocs.io/en/latest/). In short, the preprocessed, ACPC-aligned echoes were first combined according to the average rate of T2* decay at each voxel across all time points by fitting the monoexponential decay, S(t) = S0e -t / T2*, using the “nlinfit.m” function in MATLAB with least-squares optimization and the initial coefficient values obtained from a linear model fit to the log of the data. From these T2* values, an optimally combined multi-echo (OC-ME) time-series was obtained by combining echoes using a weighted average (WTE = TE * e -TE/ T2*). The covariance structure of all voxel time-courses was used to identify major signals in the resultant OC-ME time-series using principal component and independent component analysis. Components were classified as either T2*-dependent (and retained) or S0-dependent (and discarded), primarily according to their decay properties across echoes following the decision tree described in 85. We found that a global influence of respiration (a T2*-dependent signal that is not of interest per se) was retained after removing S0-dependent components. Mean gray matter time-series regression was subsequently performed to remove this spatially diffuse noise. Finally, temporal masks were generated for censoring high motion time-points using a frame-wise displacement (FD) threshold of 0.3 mm and a backward difference of two TRs (2.77 s), for an effective sampling rate comparable to historical FD measurements (approximately 2 to 4 s). Prior to the FD calculation, head realignment parameters were filtered using a stopband Butterworth filter (0.2 - 0.35 Hz) to attenuate the influence of respiration.

Child, ABCD: First, a respiratory filter was used to improve FD estimates calculated in the volume ("vol") stage. Second, temporal masks were created to flag motion-contaminated frames using the improved FD estimates. Frames with a filtered FD>0.3mm were flagged as motion-contaminated for nuisance regression only. After computing the temporal masks for high motion frame censoring, the data were processed with the following steps: (i) demeaning and detrending, (ii) interpolation across censored frames using least squares spectral estimation of the values at censored frames so that continuous data can be (iii) denoised via a GLM with whole brain, ventricular, and white matter signal regressors, as well as their derivatives. Denoised data were then passed through (iv) a band-pass filter (0.008 Hz<f<0.10 Hz) without re-introducing nuisance signals or contaminating frames near high motion frames.

Macaque: Nuisance regression using white matter (WM), and cerebrospinal fluid (CSF) signal and Friston-24 parameter models, bandpass filtering (0.01–0.1 Hz), detrending.

HCP, UKB: Resting state fMRI data were denoised for spatially specific temporal artefacts (for example, subject movement, cardiac pulsation, and scanner artefacts) using the ICA+FIX approach, which includes detrending the data and aggressively regressing out 24 movement parameters

WU120: Temporal masks were created to flag motion-contaminated frames. Motion contaminated volumes were identified by frame-by-frame displacement (FD), calculated as the sum of absolute values of the differentials of the 3 translational motion parameters and 3 rotational motion parameters. Volumes with FD>0.2 mm, as well as uncensored segments of data lasting fewer than 5 contiguous volumes, were flagged for removal. After computing the temporal masks for high motion frame censoring, the data were processed with the following steps: (i) demeaning and detrending, (ii), multiple regression including: whole brain, ventricular and white matter signals, and motion regressors derived by Volterra expansion, with censored data ignored during beta estimation, (iii) interpolation across censored frames using least squares spectral estimation of the values at censored frames so that continuous data can be passed through (iv) a band-pass filter (0.009 Hz<f<0.08 Hz) without contaminating frames near high motion frames. Censored frames were then excised from the data for all subsequent analyses.

Volume censoring: see above

Statistical modeling & inference

Model type and settings: For task data, we employed a mass univariate approach. To compute the overall degree of activation in response to each motion, data from each run was entered as a fixed effect into a first-level analysis within FSL's FEAT in which each condition timecourse was convolved with a hemodynamic response function to form a separate regressor in a GLM analysis testing for the effect of the multiple condition regressors on the timecourse of activity within every vertex/voxel in the brain. Beta value maps for each condition were extracted for each run and entered into a second-level analysis, in which run-level condition betas were tested as random effects.

Effect(s) tested: In the movement task battery, run-level condition betas were tested against a null hypothesis of zero activation in a one-sample t-test across runs (within participant).

In the action control task, a t-test across runs contrasted the run-level planning betas against the run-level execution betas (within participant).

Specify type of analysis: ☐ Whole brain ☒ ROI-based ☐ Both

Anatomical location(s): Individual-specific M1 and CON ROIs were created using each subject's resting-state fMRI data.

Statistic type for inference (See Eklund et al. 2016): ROI-wise t-values converted to Z-scores were used for inference.

Correction: Multiple comparisons were controlled using FDR correction.

Models & analysis

n/a	Involved in the study
☐	☒ Functional and/or effective connectivity
☐	☒ Graph analysis
☒	☐ Multivariate modeling or predictive analysis

Functional and/or effective connectivity: For each single-participant dataset, a vertex/voxelwise functional connectivity matrix was calculated from the resting-state fMRI data as the Fisher-transformed pairwise correlation of the timeseries of all vertices/voxels in the brain. In the ABCD, WashU120, eLABE, and PRIME-DE datasets, vertex/voxelwise group-averaged functional connectivity matrices were constructed by first calculating the vertex/voxelwise functional connectivity within each participant as the Fisher-transformed pairwise correlation of the timeseries of all vertices/voxels in the brain, and then averaging these values across participants at each vertex/voxel.

Graph analysis: To define the somatomotor regions that were visually identified from the seed-based connectivity analysis in an unbiased fashion for further exploration, we entered each individual adult human participant's data into a data-driven network detection algorithm designed to identify network subdivisions that are hierarchically below the level of classic large-scale networks (e.g. that produce hand/foot divisions in somatomotor cortex).

In each adult participant, this analysis clearly identified network structures corresponding to motor

representation of the foot, hand, and mouth; and it additionally identified network structures corresponding exactly to the previously unknown connectivity pattern identified from the seed-based connectivity exploration as the inter-effector regions. For simplicity, we manually grouped all inter-effector subnetworks together as a single putative network structure (labeled as inter-effector) for further analysis.
Finally, to identify classic large-scale networks in each participant, we repeated the Infomap algorithm on matrices thresholded at a series of denser thresholds (ranging from 0.2% to 5%), and additionally identified individual-specific networks corresponding to the Default, Medial and Lateral Visual, Cingulo-Opercular, Fronto-Parietal, Dorsal Attention, Language, Salience, Parietal Memory, and Contextual Association networks following procedures described in 32.
